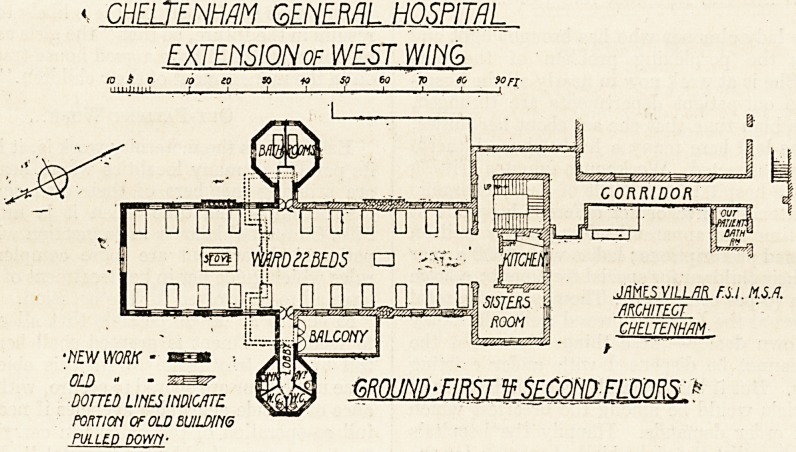# Cheltenham General Hospital

**Published:** 1910-07-23

**Authors:** 


### CHELTENHAM GENERAL HOSPITAL.

The extension to this hospital which we illustrate to-day consists of the addition of ten beds to a ward formerly containing fourteen, together with projecting octagonal wings containing bathrooms and sanitary offices. With regard to the ward itself there is nothing to be said; it is the usual type.

The sanitary offices, instead of being projected at the further end of the ward, are entered from near the centre, and for some reason or another have been planned in an octagonal form. It will readily be seen from the plan that this form does not lend itself at all well to the requirements of a sanitary block: in particular the entrance to the bathroom is extremely awkward, and it seems fairly certain that a patient could not be carried in to either bathroom. The sink-room is, as so often happens, far too small for the work that has to be done. The plan indicates an open lobby between the ward and the sanitary offices, but no suggestion of any doors, and the window in the lobby to the closets in unnecessarily small.

A balcony is provided on one side of the ward, and from the upper wards a fire-escape staircase is constructed in connection with this balcony.

The architect for this work is Mr. James Villar, F.S.I.

## Figures and Tables

**Figure f1:**